# Beyond knowing nature: Contact, emotion, compassion, meaning, and beauty are pathways to nature connection

**DOI:** 10.1371/journal.pone.0177186

**Published:** 2017-05-09

**Authors:** Ryan Lumber, Miles Richardson, David Sheffield

**Affiliations:** 1 School of Applied Social Sciences, De Montfort University, Leicester, United Kingdom; 2 Department of Life Sciences, College of Life and Natural Sciences, University of Derby, Derby, United Kingdom; University of Melbourne, AUSTRALIA

## Abstract

Feeling connected to nature has been shown to be beneficial to wellbeing and pro-environmental behaviour. General nature contact and knowledge based activities are often used in an attempt to engage people with nature. However the specific routes to nature connectedness have not been examined systematically. Two online surveys (total n = 321) of engagement with, and value of, nature activities structured around the nine values of the Biophila Hypothesis were conducted. Contact, emotion, meaning, and compassion, with the latter mediated by engagement with natural beauty, were predictors of connection with nature, yet knowledge based activities were not. In a third study (n = 72), a walking intervention with activities operationalising the identified predictors, was found to significantly increase connection to nature when compared to walking in nature alone or walking in and engaging with the built environment. The findings indicate that contact, emotion, meaning, compassion, and beauty are pathways for improving nature connectedness. The pathways also provide alternative values and frames to the traditional knowledge and identification routes often used by organisations when engaging the public with nature.

## Introduction

There is a growing realisation that a positive, connected relationship with nature leads to pro-environmental attitudes and wellbeing benefits [1,2,3]. Having a positive relationship with nature is an important part of wellbeing, comparable to established factors such as income and education [4]. Just as individual benefits to wellbeing are important, nature connectedness can also be beneficial to wider nature, as it is thought to lead to pro-environmental attitudes and subsequent positive behaviours through a willingness to sacrifice [5]. While the relationships between connectedness with nature, wellbeing and pro-environmental attitudes have been demonstrated, the specific routes to connectedness are still unclear; a full investigation into the actions and practices that lead to a connected relationship is required [6]. This is especially important given that large conservation charities in the United Kingdom (UK) including the Royal Society for the Protection of Birds (RSPB), The Wildlife Trusts and the UK branch of the World Wildlife Fund are increasingly aware of connection with nature. Further, where organisations have used knowledge and identification of species in the past as a way to engage people with nature, there is now an increasing focus on the best frames and values to use in order to engage the public [7]. Understanding the factors that facilitate increased connection to nature will complement these endeavours and inform moves to increase nature connection for both nature’s and human’s wellbeing. The present paper identifies the indicators of nature connection before operationalising them as pathways to increase connection with nature. These indicators go beyond activities that simply engage people with nature through knowledge and identification.

Being able to identify the different indicators associated with nature connection is also a good starting point for developing a theoretical account of the pathways to connectedness. Nature connection is subjective, formed through individual experiences [8,9], making the development of a theoretical account of the pathways potentially problematic, however a suitable starting point exists within the Biophilia Hypothesis [10]. The nine values of biophilia describe how humanity affiliates with nature (see Table 1). Biophilia has been suggested to function as the innate biological driver for the desire to connect with nature, for the benefits to wellbeing that nature provides [9]. As such, the nine values of biophilia and engagement activities associated with them serve as a suitable starting point for a systematic investigation of the indicators of, and pathways to nature connectedness.

**Table 1:** The nine values of biophilia [15].

Value	Definition	Function
Utilitarian	Practical use of material nature	Sustaining physical life and security
Naturalistic	Pleasure from contact with nature	Development of mental, physical and outdoor skills and development
Ecologistic-Scientific	Scientific study of the interconnectedness of nature and natural systems	Observing nature, increasing knowledge and understanding
Aesthetic	Appeal of nature’s physical beauty	Feelings of security, inspiration and contentedness
Symbolic	Expressing ideas through nature based language and metaphors	Developing mentally, communicating with others/nature
Humanistic	Emotional bond with, and love for nature	Companionship, bonding and co-operation
Moralistic	Ethical concern/judgements and revering nature	Moral reasoning, meaning of life, affiliation
Dominionistic	Control and dominance of nature	Technological/mechanical skill, physicality, control
Negativistic	Aversion, removal and fear of nature	Security and physical protection

### The Biophilia Hypothesis

The human species evolved in the savannahs of Africa over 200,000 years ago and subsequently migrated across the Earth and survived in a range of climates [11]. The landscape was important for survival as it offered both opportunities and threats, leading to preferences for certain aspects of nature and aversion to others [12]. Humanity has thus been shaped both cognitively and emotionally over time through interactions with nature [13], leading to the development of a need and desire to affiliate with life or lifelike processes known as the Biophilia Hypothesis [14,15]. The emotional bond expressed unconsciously through biophilia leads to a reverence for nature that incorporates awe and wonder, with this reverence creating a love for life and the complexity of nature [16]. It has been suggested that this affiliation or love for life was essential for survival and often sought after by humanity’s ancestors [15,17]. The innate biological tendencies of biophilia enable humanity to easily learn how to interact with nature [13] although it is now thought this is achieved through experiential learning rather than the product of an innate genetic transmission [18]. The expression of an affiliation for life is often unconscious, manifesting in art, ethics, cognitions, and emotions [14], taking place through the nine biophilic values.

This unconscious affiliation with life may explain the preferences children have for savannah-like environments [19] and visitor attendance at zoos being higher than all of the sporting events combined in the U.S.A. [15]. The Biophilia Hypothesis is not without critique as the ambiguous nature of the theory makes the direct testing of the rubrics of biophilia difficult [20]. Whether it is down to a lack of evidence or inability to test the hypothesis directly, research into biophilia has declined, however the hypothesis remains a useful catalyst for research into the human-nature relationship [21]. Biophilia has been drawn upon in the creation of two measures of connectedness with nature; the Nature Relatedness scale [22] and the Love and Care for Nature scale [16]. More recent qualitative work has utilised the nine values of biophilia as a framework for investigating the specific interactions with nature that act as routes to nature connectedness [23].

### Nature connectedness

The human-nature relationship is guided by perceptions of self and how at both a species and a personal level, humanity fits into the wider natural environment [24]. A prevailing view held by Western, often industrialised societies is that humanity is set apart from [25] and even above nature [26]. A disconnected relationship with nature is a consequence of an anthropocentric viewpoint that gained popularity as a result of the scientific revolution that took place between the 16^th^ and 17^th^ centuries [27,28]. As a result, anthropocentrism quickly became part of prevailing Western thought through a process of unconscious socialisation [27]. It is estimated that globally, humanity now dwells more within urban rather than rural locations [4] and where once affiliating with nature directly afforded survival opportunities [13,14,15] this may no longer be the case, causing a further disconnection with nature to occur through the extinction of nature experiences [29]. Nature experiences are not entirely lost however, as the presence of nature within urban environments offers an opportunity to reconnect with nature [30,31].

Reconnecting humanity with nature has become an increasing focus for research into the human-nature relationship [16,23,31,32].For this to be achieved, an extension of an individual’s concept of self to include nature is necessary. While a variety of terms have been used to describe a personal connection with nature, each focussing on the human-nature relationship from a different perspective, all describe what is essentially the same construct; nature connectedness [4,33]. A connection with nature creates a sense of belonging to the wider natural world as part of a larger community of nature [34]. Nature connectedness is therefore an appreciation and value for all life that transcends any objective use of nature for humanity’s purposes [22]. This does not mean that connectedness is the same for everyone as it is a subjective and multidimensional construct [2,33]. Nature connectedness is subject to personal and social influences [35] and is comprised of cognitive [36], affective [37], learnt, experiential [22] and personality factors [38] that together, create a connection with nature.

### Biophilia and the possible routes to nature connectedness

Research into nature connectedness has placed an emphasis on direct experiences with nature that lead to an affective and/or cognitive relationship to form [39]. When engaging with nature, the type of activity chosen may be vital to whether nature is perceived positively and if a connection is formed; thereby leading to repeated engagement with nature and facilitation of connectedness, with its associated benefits to humanity’s and nature’s wellbeing. Environmental connectedness (a sense of belonging to the natural landscape similar to nature connection) is positively related to engaging with nature through bird watching and gardening [40]. Outdoor pursuits such as hiking and camping [41] and walking in natural settings [34] are also suggested to lead to an increased connectedness with nature. The aforementioned activities include elements of a physical engagement with nature and so mirror the naturalistic value of Biophilia that places an emphasis on outdoor skill development and contact [10].

An emotional attachment to nature may also form through the anthropomorphising of nature. Anthropomorphism may be important for including nature within the self as it is the ‘cognitive mechanism’ for developing a biocentric ethos [28]. As natural elements are humanised, feelings of similarity and empathy are formed [42]. This emotional attachment to nature is also crucial to the formation of connectedness and feeling part of the natural world [37], similar to the humanistic value of biophilia; an attachment for nature born out of a love for life often through an attachment to animals [14]. Such emotional attachments to nature may also be influenced by childhood exposure to nature [38], which endures as a stable trait or subjective connection to nature [4]. While childhood experiences are important, anthropomorphism can still act as a route to connectedness with nature in adult populations [42] further indicating that childhood exposure is but one possible (albeit, important) route to nature connectedness.

Beyond a love for animal life, humanity has a preference for aesthetically pleasing nature [43] which is unsurprising given that from an evolutionary viewpoint, survival would occur by directing a large amount of attentional resources to the visual cues within the environment that would be aided by an affiliation with life [4]. This may explain why the aesthetics of nature have been linked to preferences for certain natural environments, [44]. Although theorised and included as a value of biophilia, there is little evidence of a direct link between aesthetics and nature connection. Indeed, more recent research suggests a nuanced relationship, with engaging with natural beauty being identified as impacting on the relationship between connection to nature and wellbeing [8], as the beauty of nature acts as a ‘good thing’ in nature [31]. This research supports alternative theories espoused by Gregory Bateson, who proposed that aesthetic experience was the route to greater connection to nature and the wider ecology [45]. Therefore, there is evidence that natural aesthetics are likely to be part of the explanation for a positive relationship between people and nature; just as aesthetics are an important part of biophilia, the visual appeal of nature is also important for connectedness with nature.

Through an observation of nature, a connected self can be realised through the use of symbolism that creates positive schemas about nature and the connected self [46]. The use of symbolism is thought to enhance the experience of a connection with nature by expanding an awareness of nature leading to a deeper relationship or connectedness [47]. While nature based symbolism is frequently used in most languages, it is no more frequent than non-animal metaphors but is still nonetheless an important value of biophilia as it is a significant part of culture [14]. Symbolism may therefore be a route to connectedness by providing a means to express the transpersonal experiences in a more than human world that connecting with nature provides [6].

Thus far, a connection with nature has been described as a cognitive and affective construct with aspects of personality and experience. A connection with nature is often seen to be responsible for the creation of an environmentally responsible individual as connectedness is linked to the possession of pro-environmental attitudes [5,37], and found to predict pro-environmental behaviour [9]. Evidence is emerging for the role threats to nature play in ‘awakening’ a personal bioethic. Such individuals describe the desire to protect nature from human threats as a facilitator of their own personal connectedness with nature [48]. Further, it has been suggested that a close identification with the diversity and interconnectedness of non-human life is a route to human sensibility and an enduring ethic and sense of direction for humanity as part of nature [19]. The conservation of nature through an ethical obligation is contained within the moralistic value of biophilia [14,10], suggesting that the desire to protect nature may not be a result of connectedness solely, but serves as a route to connectedness in its own right.

The use, control or avoidance of nature that the utilitarian, dominionistic and negativistic values entail, lay the foundation for the dominion over and exploitation of nature [14]. This is contrary to an equal value for all life emphasised by a connection with nature [49], making it unlikely that activities involving a use of or avoidance of nature will lead to nature connection. Engagement activities that focus on knowledge and an identification of nature, similar to the ecological-scientific value, have been used in attempts to encourage a connection to nature [50,51]. While the investigation of the natural world does not explicitly advocate mastery over nature, the lack of evidence of sustained increases in connection with nature through environmental education programmes suggests activities purely focussing on knowledge and identification may not be pathways to nature connectedness. This was recently supported in a study where creative arts based activities, rather than educational nature trails, were associated with increases in implicit nature connectedness [52]. It should be noted that recent research claiming that species knowledge is important in connecting people to nature, did not use a validated measure of connection with nature [53].

### Summary

While the links between connectedness with nature, wellbeing and pro-environmental attitudes have been examined, the specific routes to connectedness are still unclear. Engaging with nature through the naturalistic, humanistic, symbolic, moralistic and aesthetic values of biophilia are proposed as likely indicators of nature connection. The positive effect of connecting with nature has been evidenced but if individuals or wider society do not walk the path to connectedness, the mutual benefits of a connected relationship cannot be realised. Firstly, the indicators of a connectedness with nature need to be investigated [6] then the indicators need to be operationalised as targets for interventions to increase nature connectedness so that humanity and the natural community to which it belongs can benefit. To this end, three research studies are presented, to establish indicators of nature connectedness and then operationalise those as pathways in an intervention study.

## Study 1

### Design

An online survey was employed to investigate the routes to nature connectedness. It was predicted that engagement activities focussed on humanistic, naturalistic, symbolic, aesthetic and moralistic values would all be correlates of nature connectedness.

### Participants and procedure

A power calculation using Gpower based on the number of predictors indicated that 166 participants were needed for the study. Participants aged 18 and over were recruited through snowball sampling via social media accounts and through the University of Derby participant recruitment microsite, creating a sample consisting of 70 students and a further 133 individuals from a non-student population. A total of 203 participants took part in the study, with age ranging from 18 to 66, with a mean age of 36.90 (13.16 SD). Participants were predominantly female (145) with 175 participants residing in the UK. Participants read a combined brief/consent form that included their right to withdraw, instructions on how to do so, and the creation of a unique identifier to ensure confidentiality when visiting the host site. Participants first entered their demographics, followed by the twenty-seven indicators presented in a random order to limit activities relating to the same biophilic value from clustering together. To limit any priming, the Nature Relatedness scale was then administered after the participants had completed the indicator section. Once all pages had been completed, a final debrief was shown, with participants given 3 weeks after completing the study to withdraw by email.

### Materials

*The Nature Relatedness scale* (NR) is a measure of an individual’s trait connection to nature. During scale development and testing, the measure was linked to biophilia and positively correlated with time spent outdoors [22]. NR contains 3 factors: self (‘I am very aware of environmental issues), experience (‘I don’t often go out in nature’) and perspective (‘I think a lot about the suffering of animals’). All 21 items are measured on a 5 point Likert scale of 1 ‘disagree strongly’ to 5 ‘agree strongly’. An average score is calculated from all 21 items (after reverse scoring) and has good reliability (α = .87).

#### Nature Indicators

One of the main critiques of biophilia is that testing the rubrics of the hypothesis directly is often difficult [20]. A deep connection to nature (such as that which the Biophilia Hypothesis would propose) should equate to a greater engagement through specific interactions with nature, leading to a greater experience of nature connectedness. One way of testing biophilia directly would be to examine the level of engagement with nature through the nine values of biophilia. Twenty-seven statements, see Table 2, were selected from an initial forty-five that were based upon the description of each of the biophilic values suggested by Kellert and Wilson [15] with content validity assessed by eight individuals with an academic knowledge of the Biophilia Hypothesis. This involved providing the original definitions of the biophilic values alongside the items which were unattached to any value and randomly ordered. The academics were tasked with assigning each of the items to a biophilic value or to no value at all, with the overall assignment made by the academics dictating which items were used for each value. The final twenty-seven statements used the nine values as a framework with three indicative statements per value. The three indicative statements of each value indicator were rated on two scales; with the aim to investigate indicators that led to nature connection that were not dependant solely on opportunities currently available to participants. The first, was a 6-point ordinal scale of how often the indicator was engaged with, ranging from 1 (never) to 6 (several times a week); the second was a 5-point ordinal scale to indicate the value placed on the indicator, ranging from 1 (no value) to 5 (very valuable). Both more active engagement and valuing are included as some people have restrictions to engaging with the indicators (e.g. they have no garden at home), and these people are an important demographic to connect with nature with increasing urbanisation. Both activities and value is that some people do have the opportunity to engage with activities (.e.g no garden at home), and this is an important demographic to connect with nature with increasing urbanisation.

**Table 2:** Post validation engagement activities relating to the values of the Biophilia Hypothesis.

Value	Indicator Statement
Utilitarian	Tending to fruit or vegetables that you intend to eat
	Catching an animal for the purpose of eating it e.g. fishing, hunting etc.
	Collecting or chopping wood for fuel
Naturalistic	Enjoying a sensory experience of nature e.g. listening to birdsong, smelling wild flowers etc.
	Going bird or nature watching for leisure rather than scientific reason’s
	Watching a sunrise or sunset for more than a minute
Ecologistic-Scientific	Finding out more about an insect or other small animal
	Studying nature with some apparatus e.g. a microscope, a nature survey, binoculars etc.
	Drawing a scientific diagram of nature e.g. the anatomy of an animal a plant cell etc.
Aesthetic	Going to a natural place just to look at it e.g. visited hills to appreciate the view
	Looking at sculptures or pictures of large animals
	Taking a photo or painted a picture of a natural view e.g. of hills, rivers etc.
Symbolic	Using nature to represent an idea
	Thinking about the meaning of natural icons e.g. the green man, mother nature etc.
	Thinking deeply about the meaning of signs within nature e.g. the first flowers of spring, the first swallow of summer etc.
Humanistic	Feeling a deep emotional attachment to wild nature
	Having a conversation with others about your thoughts and feelings about nature
	Thinking about an animal you know when you are not with it e.g. at work**
Moralistic	Making ethical food or product choice e.g. free range eggs
	Being moved by a programme on animal welfare e.g. the great fish fight, intensive farming etc.
	Thinking about the treatment of nature e.g. animal welfare, protecting greenbelt land*
Dominionistic	Going rock climbing or caving
	Using vehicles in a natural place for sport e.g. quad biking, cross country driving, motocross
	Controlling pests within your garden or other green-space**
Negativistic	Staying in town rather than visiting a local park or green-space
	Using a computer rather than a green space for leisure
	Avoiding areas of wilderness or woodland

*Denotes an item removed to increase reliability as indicated by the Cronbach alpha when engaging with the Biophilic activities

**Denotes an item removed to increase reliability as indicated by the Cronbach alpha when engaging and valuing the Biophilic activities

### Ethics statement

All participants provided informed written consent. The Psychology Research Ethics Committee at the University of Derby approved the study and consent procedure.

## Results

### Reliability of the two nature indicator measures

The indicator measures were examined using Cronbach’s alpha to determine the reliability of the nine scales with each initially consisting of three items. While a cut-off of .7 is widely reported as an acceptable reliability, a set alpha level for acceptability does not exist given that levels perceived as low can still be useful indicators [54]. Instead, context is more important [55] and it was decided by the authors that any indicator meeting a .5 threshold would be acceptable and included in the analysis in order to cover as much of the range of human interactions with nature that biophilia provides; and to avoid missing any potential pathways to nature connectedness. The engagement with indicator for aesthetic (α = .63), naturalistic (α = .71), utilitarian (α = .32), negativistic (α = .54), ecologistic-scientific (α = .64) and symbolic (α = .76) could not be improved through the removal of any items. A single item was removed to increase the reliability of three of the indicators. The three indicators had the following Cronbach alphas: dominionistic (α = .38), humanistic (α = .83) and moralistic (α = .57).

The valuing the nature engagement indicator for aesthetic (α = .64), naturalistic (α = .73), utilitarian (α = .49), negativistic (α = .53), ecologistic-scientific (α = .74), symbolic (α = .78) and moralistic (α = .53) could not be improved through the removal of any items. A single item was removed to increase the reliability of the following indicators: dominionistic (α = .47) and humanistic (α = .78). Given that the indicators for the utilitarian and domionistic values had Cronbach’s alphas that were below .5 and could not be improved to meet this threshold through the removal of any items it was decided to exclude the indicators from the analysis. The mean scores for nature connectedness and for each of the biophilia indicators are presented in Table 3.

**Table 3:** The mean engagement scores for the engaging with and valuing indicators (N = 203).

Nature Connectedness	Mean	Minimum	Maximum	Range
	3.80 (.62 *SD*)	2.05	5.00	2.95
Biophilic Indicator	Mean Score			
	Engaging		Valuing	
Aesthetic	4.10 (1.04 *SD*)		3.69 (.79 *SD*)	
Naturalistic	3.92 (1.18 *SD*)		3.70 (.94 *SD*)	
Negativistic	3.51 (1.12 *SD*)		2.25 (.81 *SD*)	
Ecologistic-Scientific	2.72 (1.15 *SD*)		2.72 (1.04 *SD*)	
Humanistic	4.14 (1.49 *SD*)		3.57 (1.09 *SD*)	
Symbolic	3.52 (1.34 *SD*)		3.39 (1.09 *SD*)	
Moralistic	4.49 (1.20 *SD*)		3.62 (.92 *SD*)	

### Nature indicators & nature connectedness correlations

Taking part in the indicators were all related to nature connectedness, with only the negativistic indicator being negatively related, with all the relationships being significant. A breakdown of the correlations can be found in Table 4. The value attached to engaging with the indicators were all related to nature connectedness, with only the negativistic indicator showing a negative relationship. All of the correlations were significant and are presented in Table 5.

**Table 4:** Inter-Correlations between valuing being able to engage with the nine values of biophilia (N = 203).

		NR	Aesthetic	Naturalistic	Negativistic	Ecologistic-Scientific	Humanistic	Symbolic	Moralistic
NR	Pearson Correlation	1	.608**	.627**	-.361**	.493**	.716**	.634**	.606**
	Sig (2-Tailed)		.000	.000	.000	.000	.000	.000	.000
Aesthetic	Pearson Correlation		1	.673**	-.240**	.579**	.726**	.637**	.538**
	Sig (2-Tailed)			.000	.000	.000	.000	.000	.000
Naturalistic	Pearson Correlation			1	-.324**	.522**	.749**	.656**	.504**
	Sig (2-Tailed)				.000	.000	.000	.000	.000
Negativistic	Pearson Correlation				1	-.238**	-.324**	-.187**	-.132
	Sig (2-Tailed)					.001	.000	.008	.060
Ecologistic-Scientific	Pearson Correlation					1	.577**	.511**	.464**
	Sig (2-Tailed)						.000	.000	.000
Humanistic	Pearson Correlation						1	.736**	.604**
	Sig (2-Tailed)							.000	.000
Symbolic	Pearson Correlation							1	.567**
	Sig (2-Tailed)								.000
Moralistic	Pearson Correlation								1
	Sig (2-Tailed)								

** Significant at p < 0.01

* Significant at p < 0.05

**Table 5:** Inter-Correlations between engaging with the nine values of biophilia (N = 203).

		NR	Aesthetic	Naturalistic	Negativistic	Ecologistic-Scientific	Humanistic	Symbolic	Moralistic
NR	Pearson Correlation	1	.537**	.615**	-.141*	.404**	.720**	.520**	.545**
	Sig (2-Tailed)		.000	.000	.045	.000	.000	.000	.000
Aesthetic	Pearson Correlation		1	.622**	-.036	.568**	.657**	.522**	.517**
	Sig (2-Tailed)			.000	.000	.000	.000	.000	.000
Naturalistic	Pearson Correlation			1	-.163*	.325**	.673**	.606**	.404**
	Sig (2-Tailed)				.020	.000	.000	.000	.000
Negativistic	Pearson Correlation				1	.122	-.061	-148*	-.030
	Sig (2-Tailed)					.084	.388	.035	.670
Ecologistic-Scientific	Pearson Correlation					1	.521**	.328**	.497**
	Sig (2-Tailed)						.000	.000	.000
Humanistic	Pearson Correlation						1	.620**	.582**
	Sig (2-Tailed)							.000	.000
Symbolic	Pearson Correlation							1	.572**
	Sig (2-Tailed)								.000
Moralistic	Pearson Correlation								1
	Sig (2-Tailed)								

** Significant at p < 0.01

* Significant at p < 0.05

### Engaging with nature indicators & nature connectedness regressions

Pedhazur [56] states that multiple regression is eminently suited for analysing effects of independent variables (engagement and valuing) on a dependent variable (nature connection) and that the choice of variables and approach should be determined by a theoretical framework (values of biophilia) that explains the nature of the relations of the variables being studied. Including multiple predictors do not necessarily mean a large effect, as they must be well chosen and measured. A multiple regression was used to test if engaging with the nine values of biophilia predicted nature connectedness. For the regression analysis, the amended items of the dominionistic, humanistic and moralistic indicators were used. Collinearity issues were checked using VIF values, which were all below 10 (average VIF = 2.28) and the tolerance statistics which were all above 0.2. This indicated that multicollinearity was not a concern. Additionally, the assumption of errors was tested with the Durbin-Watson, which met the assumption of independent errors (Durbin-Watson = 2.37). The multiple regression indicated the nine values explained 61% of the variance of nature connectedness (R^2^ = .61, *F*(7, 195) 42.88, *p* = 0.01). The humanistic (β = .28, *p* = .002), symbolic (β = .15, *p* = .038), moralistic (β = .24, *p* = .001) and negativistic (β = -.16, *p* = .001) values were all significant predictors of nature connectedness. The aesthetic (β = .08, *p* = .270), naturalistic (β = .08, *p* = .268), and ecologistic-scientific (β = .01, *p* = .824) were not significant predictors of nature connectedness in the model.

### Valuing nature indicators & nature connectedness regressions

A multiple regression was used to test if valuing being able to engage with the nine values of biophilia predicted nature connectedness. For the regression analysis, the amended items of the dominionistic and humanistic indicators were used. Collinearity issues were checked using VIF values, which were all below 10 (average VIF = 2.04) and the tolerance statistics which were all above 0.2, indicating that multicollinearity was not a concern. The assumption of errors was also tested which met the assumption of independent errors (Durbin-Watson = 2.28). The multiple regression indicated the nine values explained 58% of the variance of Nature Relatedness (R^2^ = .58, *F*(7, 195) 38.02, *p* = .001). The naturalistic (β = .23, *p* = .001), humanistic (β = .46, *p* = .001) and moralistic (β = .19, *p* = .003) values were all significant predictors of nature connectedness. The aesthetic (β = -.01, *p* = .930), negativistic (β = -.08, *p* = .117), ecologistic-scientific (β = .02, *p* = .802) and symbolic (β = -.03, *p* = .666) values were not significant predictors of nature connectedness in the model.

### Mediation analysis

The aesthetic value of biophilia was found not to be a significant independent predictor of nature connectedness in the regression model. As discussed, previous theories have suggested that paying attention to natural aesthetics (and therefore being connected to nature) is important [10], but that relationship may be indirect with aesthetics interacting to bring about the benefits associated with nature connection [8]. For this reason, a mediation analysis was conducted on the significant predictors of nature connectedness from the regression analyses, with the aesthetic value as a mediator; indirect relationships were expected. Bootstrapping was used to test whether the true indirect effect would be zero using 5,000 bootstrap re-samples at a 95% confidence interval as this method has more power than the Sobel or causal steps tests [57]. The bootstrap indirect effects can be found in Tables 3 and 4. The aesthetic value was a mediator between the humanistic, symbolic and moralistic values when engaging with the indicators on the one hand, and nature connectedness on the other (see Table 6). It was also a mediator between the naturalistic and moralistic values when valuing being able to engage with the indicators on the one hand and nature connectedness on the other; it was not a significant mediator of the humanistic-nature connectedness relationship (see Table 7).

**Table 6:** Simple mediation of the indirect effects of engaging with humanistic, moralistic and symbolic values on nature connectedness (N = 203; 5000 bootstrap samples).

	B	*SE*	*T*	*p*
Humanistic to NR	.29	.02	14.54	= .001
Humanistic to Aesthetic	.51	.03	14.98	= .005
Aesthetic to NR	.12	.04	2.63	= .001
			LL95%CI	UL95%
Effect			.01	.10
	B	*SE*	*T*	*p*
Symbolic to NR	.29	.03	11.62	= .001
Symbolic to Aesthetic	.50	.04	11.72	= .001
Aesthetic to NR	.20	.04	5.15	= .001
			LL95%CI	UL95%
Effect			.06	.14
	B	*SE*	*T*	*p*
Moralistic to NR	.31	.03	10.77	= .001
Moralistic to Aesthetic	.47	.05	9.05	= .001
Aesthetic to NR	.23	.04	6.55	= .001
			LL95%CI	UL95%
Effect			.07	.15

**Table 7:** Simple mediation of the indirect effects of valuing humanistic, naturalistic and moralistic values on nature connectedness (N = 203; 5000 bootstrap samples).

	B	*SE*	*T*	*p*
Humanistic to NR	.41	.03	14.69	= .001
Humanistic to Aesthetic	.48	.04	12.37	= .043
Aesthetic to NR	.09	.05	1.72	= .001
			LL95%CI	UL95%
Effect			-.01	.09
	B	*SE*	*T*	*p*
Naturalistic to NR	.40	.04	11.06	= .001
Naturalistic to Aesthetic	.52	.05	11.26	= .001
Aesthetic to NR	.19	.05	3.64	= .001
			LL95%CI	UL95%
Effect			.04	.16
	B	SE	T	p
Moralistic to NR	.37	.04	9.22	= .001
Moralistic to Aesthetic	.45	.05	8.57	= .001
Aesthetic to NR	.27	.05	5.37	= .001
			LL95%CI	UL95%
Effect			.07	.17

## Discussion

Engaging with the humanistic, moralistic and symbolic indicators emerged as significant predictors of nature connectedness, while the value of engaging with humanistic, moralistic and naturalistic nature indicators were also significant predictors of nature connectedness. The aesthetic value acted as a mediator when engaging with humanistic, symbolic and moralistic indicators and when valuing engaging with naturalistic and moralistic indicators and nature connectedness. Due to the low Cronbach alphas found for two of the significant predictors within the regression model, a replication was conducted in study 2, investigating only the significant predictors using amended scales for the humanistic and moralistic values. The indicators for the dominionistic and utilitarian values had low Cronbach alphas and were excluded from the analysis. As the two values focus on exploitation and use of nature and are therefore not compatible with the construct of nature connectedness [49] amended indicators were not created for study 2. While it has been thought that knowledge about nature can encourage a connectedness with nature [51], the ecologistic-scientific value was not a significant predictor of nature connectedness and while the indicator showed good reliability (α = .64) the value was also not included in study 2.

## Study 2

### Design

A replication of study 1 was conducted again using indicators to measure the humanistic, moralistic, symbolic, naturalistic, aesthetic and negativistic values of biophilia. It was predicted that the humanistic, symbolic, moralistic and naturalistic values would all be significant predictors of nature connectedness with aesthetics once again acting as a mediator.

### Participants and procedure

The large effect size obtained in the first study suggests the measures created for study 1 have been determined correctly and explain a notably large variance in nature connection. In order to detect an effect size of R^2^ = .61, obtained in the previous study, a sample size of at least 64 participants (power of .8 and alpha of .05) was targeted. A total of 118 participants took part in the replication study, 79 were female with a mean age of 38.76 (15.32 *SD*), ranging from 18–78 years with 104 participants residing in the UK. All participants were recruited through snowball sampling via social media accounts. Study 2 followed the same procedure outlined for study 1, with participants being unable to take part if they had already engaged in the first study.

### Materials

Alongside the Nature Relatedness scale [22] the indicator items used to measure the engagement with symbolic (α = .83), naturalistic (α = .78), and aesthetic (α = .64) values of biophilia in study 1 were once again used. The negativistic indicator had a single item removed to improve its reliability (α = .53). Measures of the valuing of engaging with symbolic (α = .75), naturalistic (α = .81), and aesthetic (α = .70) indicators were also included. The negativistic indicator could not be improved through the removal of an item (α = .46) and was excluded from the analysis. Both the humanistic and moralistic values had an item removed based on the Cronbach’s alpha obtained in study 1 and replaced with a new item (see Table 8). The resulting scales for engaging with humanistic (α = .79) and moralistic (α = .59) indicators and the value of engaging with the humanistic (α = .83) and moralistic (α = .72) indicators showed greater reliability than in study 1.

**Table 8:** Replacement biophilia activity items used in study 2.

Biophilic Value	Study 1 Item	Study 2 Replacement Item
Humanistic	Thinking about an animal you know when you are not with it e.g. at work	Loved being in nature
Moralistic	Thinking about the treatment of nature e.g. animal welfare, protecting greenbelt land	Displayed a moral responsibility towards nature

### Ethics statement

All participants provided informed written consent. The Psychology Research Ethics Committee at the University of Derby approved the study and consent procedure.

## Results

The mean scores for nature connectedness and for each of the biophila indicators are presented in Table 9.

**Table 9:** The mean engagement scores for the engaging and valuing indicators (N = 118).

Nature Connectedness	Mean	Minimum	Maximum	Range
	3.96 (.60 *SD*)	1.16	5.00	3.24
Biophilic Indicator	Mean Score			
	Engaging		Valuing	
Aesthetic	4.27 (1.04 *SD*)		3.72 (.88 *SD*)	
Naturalistic	4.35 (1.28 *SD*)		4.03 (.99 *SD*)	
Negativistic	3.64 (1.36 *SD*)		Excluded from the Analysis	
Humanistic	4.72 (1.19 *SD*)		4.03 (.95 *SD*)	
Symbolic	3.75 (1.36 *SD*)		3.37 (1.13 *SD*)	
Moralistic	4.78 (1.05 *SD*)		4.06 (.87 *SD*)	

### Nature indicators & nature connectedness correlations

Taking part in the indicators were all significantly related to nature connectedness, with only the negativistic indicator having a negative relationship. A summary of the correlations is presented in Table 10. The value attached to engaging with the indicators were all positively related to nature connectedness, (r = .56) and were significant. A summary of the correlations is presented in Table 11.

**Table 10:** Inter-Correlations between engaging with the six biophilic values (N = 118).

		NR	Aesthetic	Humanistic	Symbolic	Moralistic	Naturalistic	Negativistic
NRTOTAL	Pearson Correlation	1	.500**	.729**	.630**	.619**	.717**	-.211*
	Sig. (2-tailed)		.000	.000	.000	.000	.000	.022
Aesthetic	Pearson Correlation		1	.661**	.494**	.477**	.659**	-.046
	Sig. (2-tailed)			.000	.000	.000	.000	.618
Humanistic	Pearson Correlation			1	.720**	.622**	.836**	-.150
	Sig. (2-tailed)				.000	.000	.000	.104
Symbolic	Pearson Correlation				1	.601**	.665**	-.029
	Sig. (2-tailed)					.000	.000	.756
Moralistic	Pearson Correlation					1	.646**	-.025
	Sig. (2-tailed)						.000	.791
Naturalistic	Pearson Correlation						1	-.204*
	Sig. (2-tailed)							.027
Negativistic	Pearson Correlation							1
	Sig. (2-tailed)							

** significant at p < 0.01

* significant at p < 0.05

**Table 11:** Inter-Correlations between valuing being able to engage with the five biophilic values (N = 118).

		NR	Aesthetic	Humanistic	Symbolic	Moralistic	Naturalistic
NRTOTAL	Pearson Correlation	1	.563**	.734**	.644**	.657**	.711**
	Sig. (2-tailed)		.000	.000	.000	.000	.000
Aesthetic	Pearson Correlation		1	.688**	.562**	.580**	.745**
	Sig. (2-tailed)			.000	.000	.000	.000
Humanistic	Pearson Correlation			1	.762**	.649**	.828**
	Sig. (2-tailed)				.000	.000	.000
Symbolic	Pearson Correlation				1	.608**	.680**
	Sig. (2-tailed)					.000	.000
Moralistic	Pearson Correlation					1	.647**
	Sig. (2-tailed)						.000
Naturalistic	Pearson Correlation						1
	Sig. (2-tailed)						

** Significant at p < 0.01

* Significant at p < 0.05

### Engaging with nature indicators & nature connectedness regressions

A multiple regression was used to test if engaging with the nine values of biophilia predicted nature connectedness. Collinearity issues were checked using VIF values, which were all below 10 (average VIF = 2.60) and the tolerance statistics which were all above 0.2, indicating that multicollinearity was not a concern. The assumption of errors was also tested which met the assumption of independent errors (Durbin-Watson = 2.05). The multiple regression indicated the six values explained 62% of the variance of nature connectedness (R^2^ = .62, *F*(6, 111) 28.77, *p* = .001) with humanistic and moralistic values significant predictors of nature connectedness within the model. Aesthetic, naturalistic, negativistic and symbolic values were not significant predictors of nature connectedness. The variables included within the model for engaging with the nature indicators for study 2 are presented in Table 12, with the β values from study 1 included for comparison.

**Table 12:** Engagement with nature indicators as predictors of nature connectedness (N = 118).

			Study 2	Study 1
	*b*	β	*p*	β
Constant	2.23		= .001	
Aesthetic	-.02	-.03	= .725	.072
Naturalistic	.11	.24	= .052	.088
Negativistic	-.03	-.07	= .249	-.160*
Humanistic	.16	.32	= .008*	.283*
Symbolic	.06	.13	= .141	.148*
Moralistic	.11	.20	= .018*	.239*

*Significant Result

A multiple regression was used to test if the value of being able to engage with the nine values of biophilia predicted nature connectedness. Collinearity issues were checked using VIF values, which were all below 10 (average VIF = 3.05) and the tolerance statistics which were all above 0.2, indicating that multicollinearity was not a concern. The assumption of errors was also tested which met the assumption of independent errors (Durbin-Watson = 1.97). The multiple regression indicated the nine values explained 64% of the variance of nature connectedness (R^2^ = .62, *F*(6, 111) 35.98, *p* = 0.01) with humanistic, moralistic, and naturalistic values significant predictors of nature connectedness within the model. The aesthetic and symbolic values were not significant predictors of nature connectedness. The variables included within the model for the value of being able to engage with nature indicators for study2 are presented in Table 13, with the β values from study 1 included for comparison.

**Table 13:** Value of engagement nature indicators as predictors of nature connectedness.

			Study 2	Study 1
	b	β	p	β
Constant	2.10		= .001	
Aesthetic	-.03	-.04	= .631	-.006
Naturalistic	.15	.25	= .038*	.230*
Humanistic	.20	.31	= .012*	.461*
Symbolic	.06	.11	= .247	-.029
Moralistic	.18	.25	= .003*	.193*

*Significant Result

### Value of nature indicators & nature connectedness regressions

#### Mediation analysis

A mediation analysis was again conducted on the significant predictors using the bootstrapping approach [57]. Aesthetics was a significant mediator of the relationship between engaging with (see Table 14), and the value of being able to engage (see Table 15) in moralistic indicators and nature connectedness.

**Table 14:** Simple mediation of the indirect effects of engaging with humanistic and moralistic indicators on nature connectedness (n = 118; 5000 bootstrap samples).

	B	*SE*	*t*	*p*
Humanistic to NR	.37	.03	11.48	= .001
Humanistic to Aesthetic	.58	.06	9.49	= .001
Aesthetic to NR	.02	.05	.38	= .353
			LL95%CI	UL95%
Effect			-.05	.07
	B	*SE*	*t*	*p*
Moralistic to NR	.35	.04	8.49	= .001
Moralistic to Aesthetic	.47	.08	5.84	= .001
Aesthetic to NR	.15	.05	3.33	= .001
			LL95%CI	UL95%
Effect			.03	.13

**Table 15:** Simple mediation of the indirect effects of valuing humanistic, naturalistic and moralistic indicators on nature relatedness (n = 118; 5000 bootstrap samples).

	B	*SE*	*t*	*p*
Humanistic to NR	.46	.04	11.63	= .001
Humanistic to Aesthetic	.64	.06	10.21	= .001
Aesthetic to NR	.08	.06	1.27	= .103
			LL95%CI	UL95%
Effect			-.03	.12
	B	*SE*	*t*	*p*
Naturalistic to NR	.43	.04	10.88	= .001
Naturalistic to Aesthetic	.66	.06	12.01	= .001
Aesthetic to NR	.05	.07	.77	= .220
			LL95%CI	UL95%
Effect			-.05	.12
	B	*SE*	*t*	*p*
Moralistic to NR	.46	.05	9.38	= .001
Moralistic to Aesthetic	.59	.08	7.67	= .001
Aesthetic to NR	.19	.06	3.32	= .001
			LL95%CI	UL95%
Effect			.04	.18

## Discussion

Both the engagement and valuing humanistic and moralistic indicators were significant predictors of nature connectedness in both studies 1 and 2. The naturalistic valuing indicator was also significant in both studies, with symbolic indicators significant in study 1 only. The aesthetic value was consistently found to act as a mediator of moralistic indicators and nature connectedness, while also being involved in the relationship between humanistic, symbolic, and naturalistic indicators and nature connection in study 1; therefore nature’s beauty may have a role in developing a connection to nature although the cross-sectional design precludes definitive assertions about it. The Biophilia Hypothesis was utilised as a framework to guide the selection of the indicators to investigate the pathways to nature connectedness. As nature connection was the focus of the research rather than the expression of the nine values of biophilia, the significant predictors have been renamed to distinguish them from the biophilic values, in the hope of facilitating their applied use and ease of understanding for the general public. The naturalistic value was renamed contact; the humanistic, emotion; the moralistic, compassion; the aesthetic, beauty; and the symbolic, meaning.

## Study 3

The two studies above present five indicators which are potential pathways to nature connectedness; contact, emotion, meaning, compassion and beauty. While these dimensions have been identified as potential pathways to nature connectedness, they require experimental testing. If contact, emotion, meaning, compassion and beauty are pathways to nature connectedness, activities based around them during engagement with nature should increase nature connection. A quasi-experimental walking intervention was therefore conducted to examine the effectiveness of the themed activities. Contact was inherent within the walking activity, while beauty was also present through the focussing of attention to the visual aesthetics of nature. For the remaining pathways, specific activities were created to represent emotion, meaning, and compassion and were used during the walk. It was predicted that walking in nature and engaging with nature via the pathways would significantly increase nature connectedness when compared to walking in nature alone or walking in and engaging with the built environment. Furthermore, it was predicted that only the two nature conditions would lead to a significant increase in vitality (a measure of wellbeing) with no significant increase shown when walking in a built environment.

### Design

A mixed design was used, with measures taken before and after one of three conditions: walking in a nature with three pathway activities, walking in nature with no pathway activities and walking in an urban environment with three pathway activities. The dependant variables were nature connectedness and vitality.

### Participants

Using the means and standard deviations obtained in previous research investigating the effect of walking in nature and built environments on connectedness with nature, a Cohen’s d of .81 was calculated [32]. The Cohen’s d was converted to an f value for use in GPower. The resulting calculation indicated a minimum of 69 participants were required to detect differences with a power of .8 and alpha of .05. A total of 72 participants (14 male) with a mean age of 23.93, ranging from 18 to 57 years old took part. The specific breakdowns for the three conditions can be found in Table 16. The participants were split evenly across the three groups with 24 participants in each condition. All were recruited via the University of Derby psychological research engagement system in exchange for engagement points for taking part.

**Table 16:** Mean age, age range and gender of the three conditions.

Condition	Mean age	Age range	Gender
Built activity	22.25 (6.44 *SD*)	18 to 45	21 Female (3 Male)
Pathway activity	22.21 (6.92 *SD*)	18 to 52	20 Female (4 Male)
Nature control	27.33 (9.38 *SD*)	19 to 57	17 Female (7 Male)

### Materials

The Nature Relatedness scale [22], the Subjective Vitality Scale [58], and the International Physical Activity Questionnaire [59] were utilised as pre and post measures. An Amazon Fire HD tablet and television screens were used to play activity related videos to the participants and paper and pens were used by participants to record their observations of meaning from the immediate environment.

### Ethics statement

All participants provided informed written consent. The Psychology Research Ethics Committee at the University of Derby approved the study and consent procedure.

### Procedure

The participants initially responded to a notice advertising a study that was investigating the use of spaces at the University of Derby on the psychological research engagement system by signing up for a participation ‘slot’. Multiple slots were available for participants to sign up to with each slot randomly assigned to one of the three conditions prior to being made available to the participants for selection. Each slot had a minimum of one and a maximum of ten participants taking part. Before attending, participants were instructed to wear suitable clothing for outdoor walking, regardless of the condition to which they had been assigned. The weather conditions present during each session and time of day were also recorded prior to the session starting. Upon arrival, participants were given an information sheet that indicated the study was investigating the use of spaces within the University of Derby and would involve answering questions and a guided walk. Any participants who suffered from mobility issues would be excluded from the study (although this did not occur). Once the participants had read the information sheet and understood what taking part entailed, they then signed the consent section agreeing to take part. Irrespective of the condition, all participants completed a questionnaire pack that contained demographics, the Nature Relatedness scale and the Subjective Vitality scale. Four different versions were used that presented the order of measures differently in order to counter balance the questionnaire pack for both pre and post guided walk. Also included within the questionnaire pack was the International Physical Activity Questionnaire, to diminish any demand characteristics. Participants in the pathway activity condition were led by the researcher on a walk around the green spaces around the exterior of the University of Derby and were told to pay attention to their surroundings while on the walk.

At three set points (the roof of a single storey building overlooking lawned areas, a wooded grass area to the rear of the University and a koi carp pond) the walk was stopped. At the set points participants engaged with either an emotion-beauty, meaning-beauty or compassion-beauty activity. The emotion-beauty activity consisted of having a conversation with others about their thoughts and feelings about the nature the participants had seen or could presently see. For meaning-beauty, participants spent five minutes writing down the meaning of any symbolism they could infer from the nature they could see or had seen on the walk. While participants watched the RSPB’s build a home for nature video for the compassion-beauty activity. In the indoor environment activity condition, participants were led on a walk around the interior of the University campus and told to pay attention to their surroundings while on the walk. Again at three set points (a sofa in the student’s union, an atrium balcony and a seated area) the walk was stopped. At the set points, participants engaged with either an emotion-beauty, meaning-beauty or compassion-beauty activity as in the nature with pathway activity condition but with a focus on the built environment instead. For example, participants spoke about the good things they could see/had seen on the walk around the university campus for the emotion-beauty activity. In the nature control condition, the participants were also told to pay attention to their surroundings and were taken on the same walk as the pathway activity condition, but instead of undertaking set activities at the three stopping points, the participants were allowed to talk amongst themselves with no set activity provided. The walks were all of a similar distance, with the nature conditions measuring 726 metres and the built walk measuring 718 metres. Each walk including activities took approximately 20 minutes. At the end of each condition, the same measures administered prior to each condition were completed once again and participants were thanked for taking part with a full debrief provided.

## Results

The mean overall Nature Relatedness score prior to taking part was 3.27 (.62 *SD*), that increased to 3.37 (.61 *SD*) after taking part. The mean NR scores for the three conditions are presented in Table 17.

**Table 17:** Means and standard deviations of the three experimental conditions.

Condition	NR Pre	NR Post	Vitality Pre	Vitality Post
Built Activity	3.09 (.55 *SD*)	3.18 (.57 *SD*)	3.57 (.66 *SD*)	3.64 (.62 *SD*)
Pathway Activity	3.28 (.52 *SD*)	3.49 (.53 *SD*)	3.87 (.92 *SD*)	4.11 (1.12 *SD*)
Nature Control	3.45 (.73 *SD*)	3.46 (.70 *SD*)	4.08 (1.05 *SD*)	4.27 (1.23 *SD*)

The data was screened for skewness, kurtosis and outliers with all falling within acceptable parameters. In addition, the demographics of gender and age were investigated due to the group differences. Gender was negatively related to pre (r = -.05, p = .653) and post (r = -.04, p = .727) Nature Relatedness, which were non-significant. Age was positively related to pre (r = .32, p < .0001) and post (r = .38, p < .0001) Nature Relatedness, which was significant. Gender was negatively related to pre (r = -.10, p = .382) and post (r = -.15, p = .226) Vitality, which were non-significant. Age was positively related to pre (r = .32, p = .007) and post (r = .33, p = .005) Vitality, which was significant. Age wasn’t controlled for within subsequent analysis as it was skewed and had outliers, thus not meeting the assumptions for ANCOVA. Therefore, a mixed measures ANOVA was conducted to determine whether there was an interaction between taking part in either the built activity, pathway activity or nature control conditions and nature connectedness scores over time.

A Mauchley’s test demonstrated that the assumption of sphericity had not been violated (*x*^2^(0) = 0.00, *p* > 0.05); it was decided that the non-corrected degrees of freedom should be used. The mixed measures ANOVA showed that there was a significant change in nature connectedness over time *F*(1, 69) = 11.68, p = .001, ω^2^ = .39, while the interaction between condition and nature connectedness over time was significant *F*(2, 69) = 3.60, *p* = .033, ω^2^ = .27. Given that the overall interaction was significant, the data was explored further. Three paired samples t-tests were conducted to investigate the effect of condition on nature connectedness. The pathway activity condition showed a significant increase in nature connectedness (*t*(23) = -3.99, *p* = .001, (one-tailed) *r* = .41) while the built activity (*t*(23) = -1.57, *p* = .065, (one-tailed) *r* = .31 and nature control (*t*(23) = -.31, *p* = .380, (one-tailed) *r* = .06) conditions did not significantly increase nature connectedness.

A further mixed measures ANOVA was conducted on changes in vitality over time. The Mauchley’s test demonstrated that the assumption of sphericity had not been violated (*x*^2^(0) = 0.00, *p* > .05). It was decided that the non-corrected degrees of freedom should be used. There was a significant change in vitality over time *F*(1, 69) = 8.22, *p* = .005, ω^2^ = .32 but the interaction between vitality over time and condition was non-significant *F*(2, 69) = .68, *p* = .509.

## General discussion

In the cross-sectional studies, humanistic and moralistic indicators were consistent predictors of nature connectedness, while valuing being able to engage via the naturalistic indicator was also significant in both studies. The symbolic indicator also showed significance with beauty involved in these relationships to nature connectedness. Together, the indicators consistently explained around 60% of the variance in nature connectedness providing a useful explanatory model. Further, the intervention study demonstrated that the indicators could then be operationalised as pathways to increase connection to nature, meeting the call for research into the specific activities that lead to connectedness [6]. A summative matrix can be found in Table 18 of the biophilic values [16] and the identified pathways. Finally, engaging with contact based activities themed around emotion, meaning, compassion, and beauty was found to increase connection to nature in the pathway activity condition only. Furthermore, while vitality increased over time, the effect of condition on vitality was non-significant.

**Table 18:** A summative matrix of the identified pathways and their corresponding biophilic values.

Biophilic Value	Definition	Pathway	Definition
Naturalistic	Pleasure from contact with nature	Contact	The act of engaging with nature through the senses
Aesthetic	Appeal of nature’s physical beauty	Beauty	The perception of aesthetic qualities including shape, colour and form that please the senses
Symbolic	Expressing ideas through nature based language and metaphors	Meaning	Using nature or natural symbolism to communicate a concept that is not directly expressed
Humanistic	Emotional bond with, and love for nature	Emotion	An affective state or sensation that occurs as a result of engaging with nature
Moralistic	Ethical concern/judgements and revering nature	Compassion	Extending the self to include nature, leading to a concern for other natural entities that motivates understanding and helping/co-operation

The finding that the pathway activity condition led to a significant increase in connection to nature is as hypothesized. It was predicted that this increase would be more than the nature control condition, but the walk in nature did not lead to an increase in nature connectedness, as might have been expected from previous research [34]. The combined results suggest that the physical walk had no effect on the increase in nature connectedness in the pathway activity condition, nor does a short walk in nature. Whereas, the enhanced contact with nature, enhanced by sensory and emotional activities, in the intervention condition was effective. The significant increase in nature connectedness in the pathway condition supports the proposal that contact, emotion, meaning, and compassion, mediated by engagement with natural beauty, are pathways to nature connectedness.

As predicted, engaging with nature through emotion [37,60], contact [34], beauty [8], compassion [48] and meaning [47] play a role in facilitating nature connectedness. The significant effect of engaging with nature via the pathways, especially in an urban environment that was used in this study, has implications for the application of the pathways used by organisations to promote engagement with nature. The results of this study suggest that it is possible to increase the sensation of nature connectedness within an urban environment [51] but that the type of interaction with nature is important, given that merely walking in a natural setting did not increase nature connectedness.

Given that wellbeing has been frequently shown as an outcome of nature connectedness, it was not surprising that vitality, the wellbeing measure most closely related to a connection with nature [4], was shown to increase significantly over time. However, there was no significant increase by condition. It was expected that an increase in wellbeing would result from walking in nature as this has been reported previously [34]. Recent research [61] has suggested the type of natural environment may affect wellbeing differently, therefore future research should consider the type of environment when investigating wellbeing through nature connection interventions.

A limitation of all three studies is that the participants were predominately female. This is unfortunate and should be addressed in future research, however it does match the gender bias of those engaging with nature connections campaigns [62]. In study 3 specifically, it should be considered whether all the participants would have found nature to be aesthetically attractive as no indicator of aesthetic preferences was taken; future research should include this aspect. It should also be noted that age correlated with vitality and nature connectedness, with the mean age of participants being higher in the nature control condition, which necessitates more rigorous control and investigation in future research. In addition, it is acknowledged that the participants in the nature control condition may have become distracted through talking to each other as no activity was prescribed; controlling this aspect in any subsequent research is required. Further, in future research, it would be useful to investigate whether engaging with the pathways of contact, emotion, meaning, beauty, and compassion would show any long-term increases in nature connectedness. Alongside this, exploring the qualitative accounts of participants when engaging with nature via the pathways would highlight the specific experiences that occur from engaging with the pathways that lead to nature connectedness that should be the focus of future research. Each pathway should also be tested in isolation to see if they have an impact individually, or whether the combination is key. Finally, the nature of the pathways fit well with recent evidence that self-reflection is important when attempting to increase nature connection [63].

Returning to the cross-sectional studies, it was surprising that the aesthetic value (beauty) did not emerge as a direct predictor of nature connectedness in either study 1 or study 2. More interestingly, engagement with nature’s aesthetics was found to consistently mediate the relationship between the moralistic (compassion) value and nature connectedness. This mediation supports recent work on the importance of engagement with natural aesthetics [8]. It appears that the appreciation of the aesthetics of nature is an important part of the relationship between the pathways and nature connectedness.

The two cross-sectional studies measured engagement with, and value of engaging with, indicators structured around the nine values of biophilia. Biophilia has not been utilised previously to investigate the routes to nature connectedness due to a lack of a valid and reliable measure of biophilia, as testing the rubrics of the hypothesis is difficult [20]. Each value was assessed through the average of three items, with five of the nine values showing good reliability when utilising all three items, with another two having improved reliability when a single item was removed. The humanistic and moralistic indicator items were improved further with the replacement of a single item for study 2 with significant predictors of nature connectedness found, as assessed by a reliable measure. The two values that did not have a scale with a reliability of .5 or above were the utilitarian and dominionistic values in study 1, and the valuing of the negativistic indicator in study 2. The absence of activities comprised of using, dominating, or avoiding nature as pathways meet theoretical expectations, given that such activities could not lead to the valuing of all life as equal members of the natural community that nature connectedness implies [49].

Although the indicators consistently explained around 60% of the variance in nature connectedness, it is acknowledged that the reliability of some indicator items was low and that the removal of indicator items due to poor reliability was not ideal. During the creation of the measure, an initial forty-five activity items were produced and validated to obtain the twenty-seven items used, with five of the original nine scales showing good reliability through further refinement and replication in study 2. However, studies 1 and 2 were not about scale development, rather about analysing the values of biophilia to see which most related to nature connectedness, allowing an intervention to be developed. The significant findings in study 3, showing nature connection can be increased through engaging with activities based on the pathway themes identified, provide strong support for the approach taken.

### Conclusion

Activities that involved contact, meaning, emotion, compassion and beauty were found to be both indicators of, then pathways towards nature connectedness. There is a need to move beyond a superficial contact with nature or focussing exclusively on knowledge and identification, when fostering a relationship with nature. The studies presented in this paper, nor other works [52], have found support for either knowledge or superficial engagement with nature functioning as pathways to nature connection. Researchers and practitioners interested in facilitating nature connectedness and its associated benefits should focus specifically on activities that involve contact, meaning, emotional attachment, or a compassionate relationship with nature that includes engaging with nature’s beauty.

## Supporting information

S1 File Dataset for study 1. (CSV)

S2 File Dataset for study 2. (CSV)

S3 File Dataset for study 3. (CSV)
